# Inhibition of histone deacetylation in 293GPG packaging cell line improves the production of self-inactivating MLV-derived retroviral vectors

**DOI:** 10.1186/1743-422X-3-27

**Published:** 2006-04-07

**Authors:** Diana E Jaalouk, Milena Crosato, Pnina Brodt, Jacques Galipeau

**Affiliations:** 1 Department of Medicine, Lady Davis Institute for Medical Research, McGill University, Montreal, Canada; 2 Department of Medicine, McGill University Health Center, McGill University, Montreal, Canada; 3 Department of Surgery, McGill University Health Center, McGill University, Montreal, Canada; 4 Division of Hematology/Oncology, Jewish General Hospital, McGill University, Montreal, Canada; 5 Department of GU Medical Oncology, Unit 1374, The University of Texas M. D. Anderson Cancer Center, P.O. Box 301439, Houston, Texas, USA

## Abstract

**Background:**

Self-inactivating retroviral vectors (SIN) are often associated with very low titers. Promoter elements embedded within SIN designs may suppress transcription of packageable retroviral RNA which in turn results in titer reduction. We tested whether this dominant-negative effect involves histone acetylation state. We designed an MLV-derived SIN vector using the cytomegalovirus immediate early enhancer-promoter (CMVIE) as an embedded internal promoter (SINCMV) and transfected the pantropic 293GPG packaging cell line.

**Results:**

The SINCMV retroviral producer had uniformly very low titers (~10,000 infectious retroparticles per ml). Northern blot showed low levels of expression of retroviral mRNA in producer cells in particular that of packageable RNA transcript. Treatment of the producers with the histone deacetylase (HDAC) inhibitors sodium butyrate and trichostatin A reversed transcriptional suppression and resulted in an average 106.3 ± 4.6 – fold (*P *= 0.002) and 15.5 ± 1.3 – fold increase in titer (*P *= 0.008), respectively. A histone gel assay confirmed increased histone acetylation in treated producer cells.

**Conclusion:**

These results show that SIN retrovectors incorporating strong internal promoters such as CMVIE, are susceptible to transcriptional silencing and that treatment of the producer cells with HDAC inhibitors can overcome this blockade suggesting that histone deacetylation is implicated in the mechanism of transcriptional suppression.

## Background

Retroviral vectors derived from C-type mammalian retroviruses are characterized by their ability to integrate into the chromosomal DNA of their target cells. For this reason, they have been a favored method of gene transfer into dividing cells in approaches where stable and sustained gene expression is desired or necessary. Conventional retroviral vectors resemble in their architecture their wild-type counterparts in that they retain *cis*-acting promoter sequences located in the 5' and the 3' long terminal repeats (LTRs) and the Ψ signal that allows the packaging of recombinant RNA into viral particles [1,2].

Many retroviral vectors employ the use of inducible or tissue-specific promoters that are incorporated into the vector design to allow for regulated or targeted gene expression. However, the transcriptional activity of an embedded promoter can be compromised by interferences from the strong enhancer and promoter machinery in the flanking retroviral LTRs [3-5]. To bypass this problem, self-inactivating retroviral vectors (SIN) have been designed whereby the viral enhancer and/or promoter sequences are deleted from the U3 region of the 3'LTR. Following reverse transcription in transduced cells, the 3' LTR deletions will be copied to the 5'LTR by template switch rendering the vector transcriptionally inactive [6-8].

SIN vectors have been successfully used to drive regulated transgene expression by inducible promoters [9,10] and to confer restricted gene expression by cell type-or tissue-specific promoters [11-15]. Additionally, the SIN configuration results in relatively safer vectors for human gene therapy applications by reducing the risk of aberrant activation of cellular oncogenes adjacent to the integrated provirus site and by minimizing the risk of production of replication competent retroviruses (RCRs) [3,16]. For these reasons, SIN vectors have beenused in many cell and gene therapy applications including vectors derived from murine leukemia virus (MLV) [17-19], and lentivirus [20,21]. Despite their desired features, SIN vectors possess a number of limitations. They can be genetically unstable [22,23] and may exhibit rescue of the U3-deletion in the 3' LTR by the intact 5'LTR due to recombination events [6,24]. To prevent such reconstitution events, hybrid 5'LTRs have been used in which the U3 is replaced by non-homologous enhancer or promoter sequences such as the cytomegalovirus (CMV) enhancer-promoter [25,26]. Moreover, SIN vectors are often associated with reduced titers which greatly limit their gene transfer efficiency [27,28]. As is the case for conventional retroviral vectors, low titers from SIN retrovectors could in part be due to transcriptional suppression of the expression of the necessary *trans-*components in packaging cells that are required for the production of the retroviral particles. Low titers from certain MLV-based SIN vectors have been also attributed to inefficient polyadenylation of the viral RNA due to extensive deletions made to the U3 region of the 3'LTR. Such deletions included the TATA box affecting the nearby R region which is implicated in polyadenylation [8,29].

We propose that interferences between elements of strong promoters incorporated within SIN retroviral vector designs and sequences in the 5'LTR can lead to suppression of retroviral RNA transcription which in turn results in reduction of SIN retrovector titers. We hypothesize that the mechanism of transcriptional suppression in SIN vectors involves the recruitment of histone deacetylases (HDACs). To test this, we designed a SIN retroviral vector whereby a deletion was made to the U3 region of Moloney murine leukemia virus (Mo-MLV) 3'LTR removing most of the enhancer machinery that is intrinsic to the retrovirus. In this SIN template, a CMV promoter replaces the U3 region in the 5'LTR and drives expression of the retrovector mRNA in transfected packaging cell lines. As an internal promoter, we used the CMV immediate early enhancer-promoter (CMVIE) to drive expression of the enhanced green fluorescent protein (EGFP) reporter in transduced cells. The CMVIE is a very potent promoter and has been typically incorporated into retroviral and lentiviral backbones to drive strong transgene expression [21,26].

Here, we show that transcription of retroviral RNA from the resultant SINCMV retrovector was suppressed in transfected 293GPG producer cells that had dramatically low titers (~10^4 ^viral particles per ml). We further demonstrate that treatment of the SINCMV retroviral producers with the HDAC inhibitors sodium butyrate and Trichostatin A (TsA) reversed the transcriptional suppression and resulted in a significant increase in the SIN retroviral titer.

## Results

### SINCMV retrovector design and increased retroviral RNA transcription with sodium butyrate treatment in transfected 293GPG producer cells

**Figure 1:**
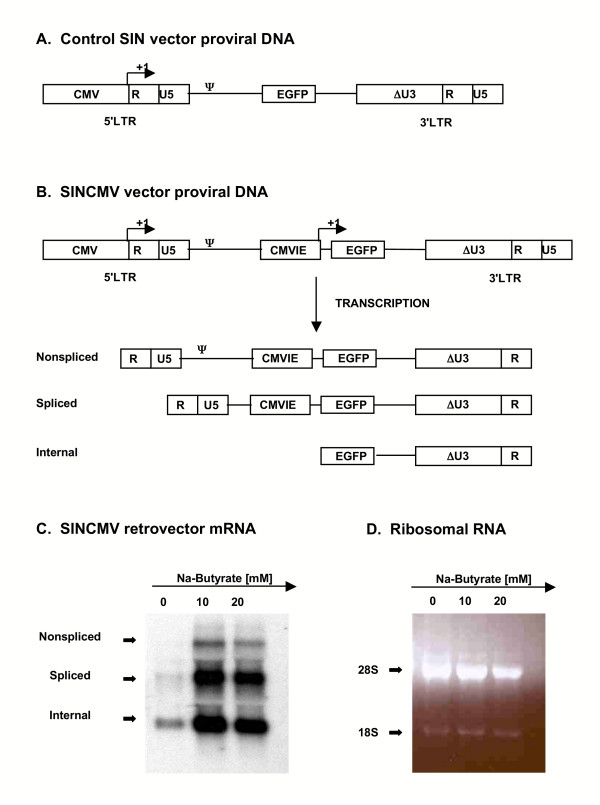
**SINCMV vector design and transcript expression in retroviral producer cells**. A. The control SIN vector lacking an internal promoter has major deletions in the 3'LTR enhancer elements rendering its intrinsic promoter machinery transcriptionally inactive in transduced cells. B. Transgene expression in the SINCMV design is driven by the internal CMVIE promoter embedded upstream of the reporter EGFP. Three RNA transcripts are expected from SINCMV proviral DNA transcription in transfected packaging cells. The upstream CMV promoter in the 5'LTR drives the expression of a full-length ~2.6 kb transcript that can be packaged into retroparticles and a ~2.1 kb spliced form that lacks the packaging signal (Ψ). The internal CMVIE drives the expression of a shorter ~1 kb transcript. C. Hybridization with a P^32^- labelled EGFP probe done on total RNA extracted from SINCMV retroviral producers treated with butyrate indicated significant increase in the level of retrovector mRNA. D. Loading control of the 3 RNA samples is shown by ribosomal RNA staining with ethidium bromide.

A SIN vector was derived from a Mo-MLV-based vector, pLTRGFP, by creating a 311-bp NheI-SacI deletion to the 3'LTR, thus removing all the retroviral enhancers and the CAAT box (Figure 1A). Then, the SINCMV vector was made by incorporating the CMVIE enhancer-promoter into the construct upstream of the cDNA for EGFP reporter to drive strong transgene expression in transduced cells (Figure 1B). In this design, the CMV promoter in the hybrid 5'LTR drives the expression of a full-length 2.6 kb RNA transcript that can be packaged into retroparticles and a ~2.1 kb spliced form that lacks the packaging signal (Ψ). The internal CMVIE drives the expression of a shorter ~1 kb transcript. Hence, retroviral RNA transcription from SINCMV proviral DNA in transfected producer cells should hypothetically result in 3 RNA transcripts, but only one is packageable (Figure 1B).

We then generated SINCMV retroviral producers by stable transfection of the 293GPG packaging cell line. The resultant polyclonal as well as isolated single clone producer populations were utilized to generate VSV-G pseudotyped retroviral particles and had very low titers in the range of ~10^4 ^viral particles per ml (see below). To test if strong promoter sequences incorporated within the SIN design can lead to suppression of retroviral RNA transcription which in turn results in reduction of SIN retrovector titers, total RNA was extracted from stable 293GPG-SINCMV producer cells, loaded onto an RNA gel, and Northern Blot analysis for the SINCMV retrovector mRNA was done using a P^32^-labeled GFP probe (Figure 1C). Expression of the 3 predicted retroviral RNA transcripts in these cells was very low with almost undetectable levels of the full-length packageable transcript from the upstream CMV promoter. However, treatment of the producer cells with 10 mM and 20 mM sodium butyrate for 48 hr resulted in a significant increase in the expression of the three transcripts and of particular importance of the non-spliced SINCMV retrovector mRNA which was undetectable in the untreated control cells. Loading of the 3 samples was controlled for by ribosomal RNA as shown by ethidium bromide imaging (Figure 1D).

### Increase in titer of SINCMV producers with sodium butyrate treatment and enhanced gene transfer into A549 cells with improved SINCMV viral titers

**Figure 2:**
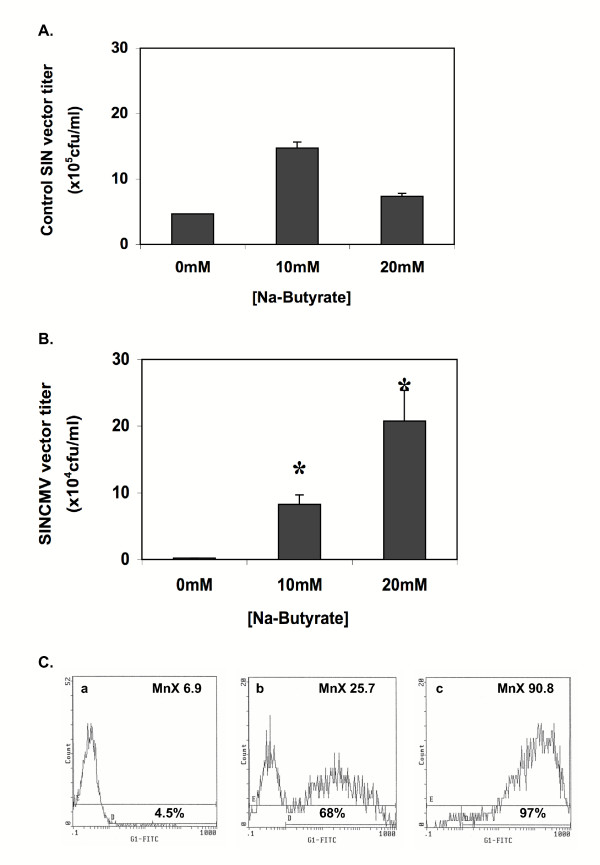
**Titer of SINCMV retroviral producers treated with sodium butyrate and transduction of A549 cells with retrovirus from butyrate treated SINCMV producer cells**. A. Treatment of control retroviral producer cells with the histone deacetylase inhibitor sodium butyrate for 48 hr resulted in a modest 1.6 ± 0.4-fold increase in titer (*P *= 0.299). B. Treatment of SINCMV retroviral producer cells with sodium butyrate for 48 hr resulted in a maximal 106.3 ± 4.6-fold increase in titer (*P *= 0.002) that was obtained with 20 mM butyrate. C. A549 lung carcinoma cells were transduced with same volume of retroviral supernatant that was collected from control-untreated SINCMV producers (a), producers treated with 10 mM sodium butyrate (b), and 20 mM sodium butyrate (c). % EGFP positive cells for each sample and mean EGFP reporter expression in the gated population (MnX) indicate a marked increase in gene transfer into target cells with supernatant from butyrate treated producers.

To determine if increased retroviral RNA transcription with sodium butyrate treatment, in particular that of the packageable retrovector transcript, would result in improved viral titer, SINCMV retroviral producers were treated with increasing doses of sodium butyrate for 48 hr, after which viral supernatants were harvested in fresh media. Following transduction of A549 cells, viral titer was measured as infectious viral particles per ml. Interestingly, 10 mM butyrate treatment resulted in a significant 42.1 ± 1.4-fold increase in viral titer (*P *= 0.001) as compared to that of control untreated producers (Figure 2B). Moreover, the increase in SINCMV retroviral titer was dose-dependent. A maximal 106.3 ± 4.6-fold increase in titer was obtained with 20 mM sodium butyrate treatment (*P *= 0.002) as determined from three independent experiments. By contrast, similar butyrate treatment of the producer cells for the control vector lacking the internal CMVIE promoter (Figure 2A) which had an average titer of ~ 5 × 10^5 ^viral particles per ml resulted in only a modest 3.2 ± 0.9-fold increase with 10 mM butyrate (*P *= 0.136) and 1.6 ± 0.4-fold increase with 20 mM butyrate (*P *= 0.299). Note that the upfront titer of the control SIN vector prior to butyrate treatment was 50-fold higher than that of the SINCMV vector.

A549 lung carcinoma cells, plated in 6-well dishes at the same number, were transduced with an equal sample volume of SINCMV retroviral supernatants collected from control untreated producers (Figure 2C, a) as well as from producers treated with 10 mM butyrate (Figure 2C, b) and 20 mM butyrate (Figure 2C, c). Flow cytometry analysis of green fluorescence on gene modified cells revealed a striking increase in gene transfer efficiency into target cells using SINCMV retroviral supernatants from 10 mM and 20 mM butyrate treated producers whereby 68% and 97% of the target cells were positive for the EGFP reporter respectively. On the other hand, only 4.5% of A549 cells were transduced with an equal volume of the control supernatant from untreated producer.

### Increased histone acetylation in SINCMV producer cells treated with sodium butyrate

**Figure 3:**
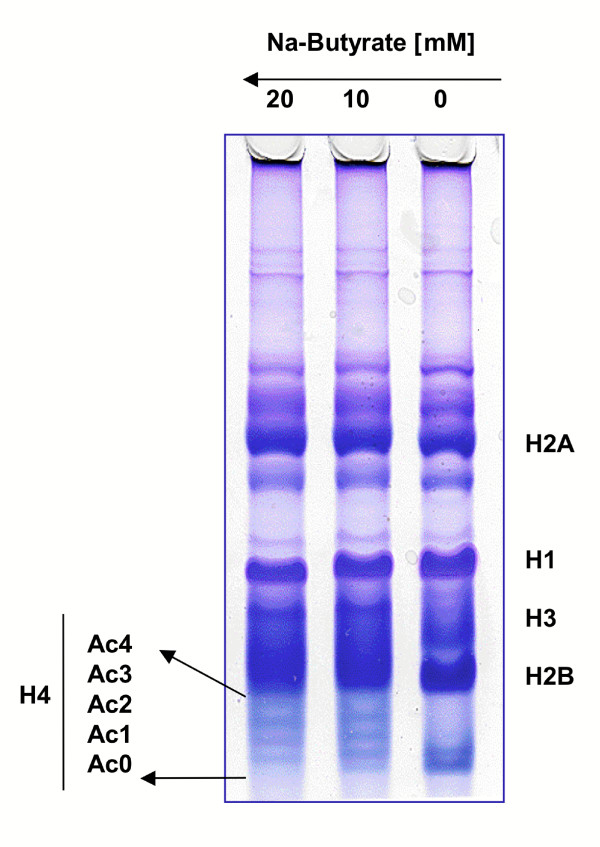
**Histone gel assay on SINCMV producer cells treated with sodium butyrate**. Treatment of SINCMV retroviral producer cells with increasing doses of sodium butyrate for 48 hr resulted in increased histone acetylation as most obvious with histone H4.

To confirm that sodium butyrate treatment resulted in increased histone acetylation in SINCMV producer cells, histone proteins were isolated from control untreated producers as well as cells treated with increasing doses of sodium butyrate. The samples were then analyzed for their acetylation status using an Acid Urea Triton gel electrophoresis (Figure 3) that separates histone proteins based on charge density in addition to size and shape, hence allowing for the detection of posttranslational modifications such as acetylation. In such a gel, the addition of an acetyl group to a lysine residue on a histone protein renders the modified protein less positive and therefore it slows down its migration in the gel. Using Histone 4 as an indicator, we observed increased acetylation of histone proteins from SINCMV producer cells that were treated with 10 mM sodium butyrate as compared to control, untreated producers. This increase in histone acetylation was even more evident in samples treated with 20 mM butyrate as can be clearly seen from the shift to greater levels of tri-acetylated and the appearance of tetra-acetylated (Ac4) histone H4 as the dose of sodium butyrate increases.

### Increase in titer of SINCMV producers with Trichostatin A treatment

**Figure 4:**
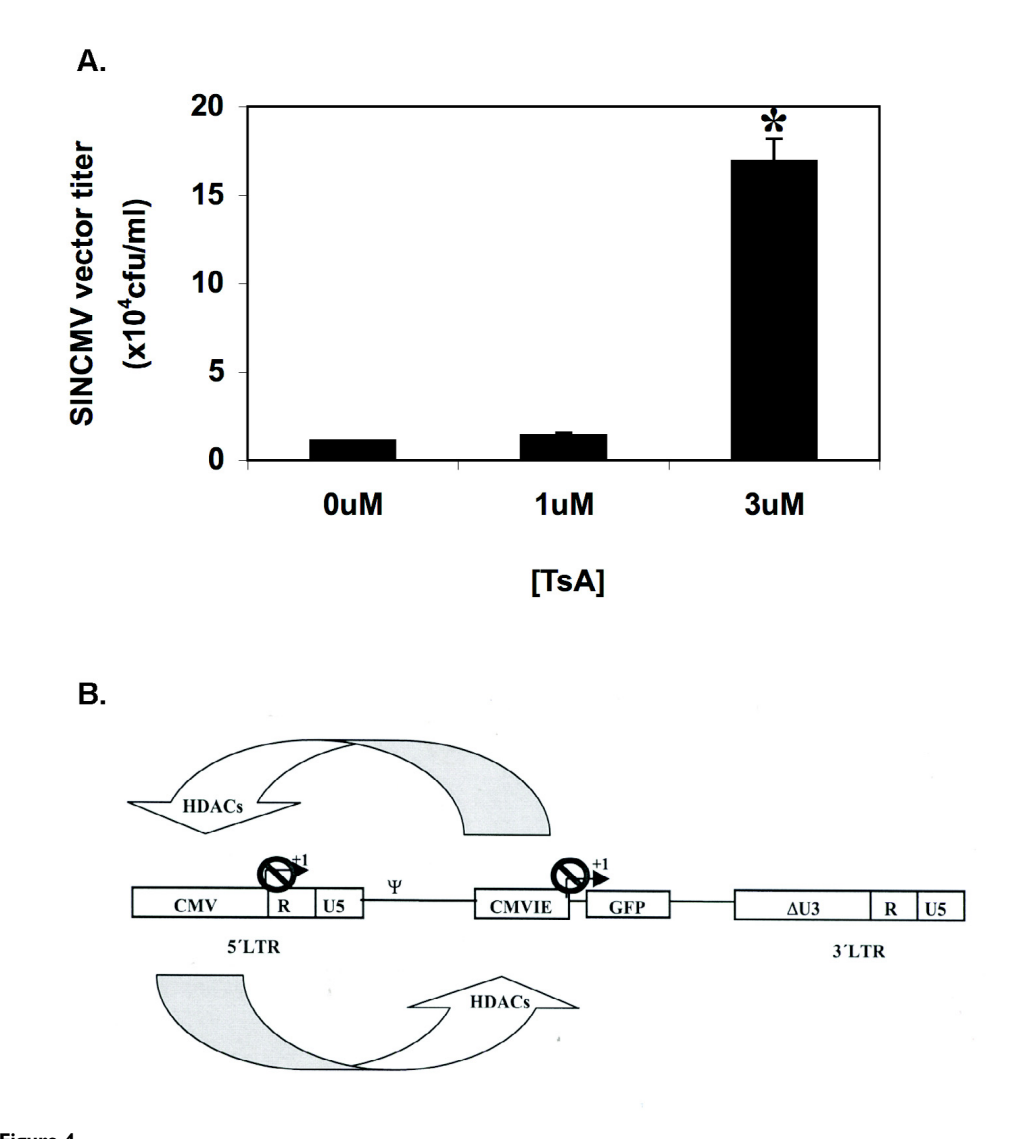
**Titer of SINCMV retroviral producers treated with TsA and model depicting mechanism of transcriptional suppression in the SINCMV design**. A. Treatment of SINCMV retroviral producer cells with the histone deacetylase inhibitor TsA for 48 hr resulted in a maximal 15.5 ± 1.3-fold increase in titer (*P *= 0.008) that was obtained with 3 μM TsA. B. Interferences between strong elements in the internal CMVIE enhancer-promoter and the upstream CMV promoter in the 5'LTR lead to the recruitment of histone deacetylases (HDACs) which trigger an inactive chromatin conformation at the promoter sites leading to transcriptional suppression of the retroviral RNA.

To determine if the resultant increase in SINCMV retroviral titers with sodium butyrate treatment is specifically due to inhibition of histone deacetylation rather than a non-specific transcriptional upregulation effect, Trichostatin A which is a potent and specific inhibitor of histone deacetylation was assessed for its effect on SINCMV retroviral titer. Treatment of the producer cells with ≤ 1 μM TsA for 48 hr did not result in any significant increase in retroviral titer (Figure 4A). However, using higher drug concentrations, an average 15.5 ± 1.3-fold increase in titer was obtained with 3 μM TsA treatment compared to control (*P *= 0.008).

## Discussion

Self-inactivating retroviral vectors are frequently designed with strong internal promoters to drive transgene expression in transduced cells, yet these designs are often associated with poor retrovector production. Low titers in the range of 10^4 ^– 10^5 ^colony-forming units per ml (cfu/ml) have been reported from SIN retrovectors that incorporated the SV40 promoter or the mouse metallothionein I (MT) promoter [6]. A dramatically low titer of ~10^3^cfu/ml was obtained from a SIN retrovector which had the TK promoter in sense orientation and the hMT inducible promoter in antisense orientation. In another study, a SIN design with an internal hybrid promoter composed of the human beta-globin promoter and CMV enhancer sequences resulted in poor gene transfer efficiency likely due to lowered titers [27]. Moreover, Mo-MLV based retroviral vectors with hybrid LTRs incorporating large portions of the melanoma-specific murine tyrosinase enhancer/promoter also had titers in the range of 10^3^cfu/ml [28]. A later study reported very low viral titers of 10^3^cfu/ml for SIN vectors in which the Mo-MLV enhancers were swapped by tandem repeats of the core element of the tyrosinase enhancer and the Mo-MLV promoter was substituted with the stronger SV40 promoter in an attempt to generate targeted retroviral vectors with higher levels of expression [30]. The authors attributed reduced titers in the latter studies to decreased efficiency of reverse transcription due to loss of a small part of the R region in the LTR. Their results also suggested a negative interference of the tyrosinase enhancer on the viral enhancer when the latter was retained in the 3'LTR. It has been also reported that the muscle creatinine kinase enhancer had a partial suppressive effect over the viral enhancer in the LTR [31]. Based on these findings, we speculated that interferences between elements of strong promoters incorporated within SIN designs and sequences in the 5'LTR can lead to suppression of retroviral RNA transcription which in turn results in reduction of titers from these vectors.

To assess the effect of promoter interferences within SIN retrovectors on viral RNA transcription and titer, we designed a Mo-MLV-based SIN vector by removing all the enhancers and the CAAT box from the 3'U3 region (Figure 1A). The TATA box and the R region were left intact to ensure efficient polyadenylation. Then, as an internal promoter, we incorporated the CMVIE enhancer promoter which is among the most potent enhancer-promoters known and has been typically incorporated into retroviral and lentiviral backbones to drive strong transgene expression [32,33]. The resultant SINCMV design (Figure 1B) which had low titers in the range of ~10^4 ^viral particles per ml has a hybrid 5'LTR in which a CMV promoter replaces the U3 region to ensure strong expression in transfected packaging cells and to minimize the risk of rescue of the SIN deletion in the 3'LTR. We expected interferences between the internal CMVIE and the upstream 5'CMV in the SIN retrovector configuration as competitive inhibition between the two promoters was previously reported in plasmid constructs [34]. Indeed, very low levels of retroviral RNA transcripts derived from both promoters were obtained in producer cells transfected with SINCMV vector (Figure 1C). The expression of the full-length packageable transcript by the 5'CMV promoter was almost completely abrogated indicating a stronger interference from the internal CMVIE. Moreover, in the absence of butyrate, we observed 50-fold higher retroviral titers in a SIN vector identical in all aspects to the SINCMV design except for the absence of the internal CMVIE promoter (Figure 2A). The sum of these observations strongly supports the notion that CMVIE is a potent *cis*-acting suppressor of promoters 5' to its location within a plasmid vector construct.

In recent years, there has been growing evidence that interference between promoter sequences are mediated by modifications to histone proteins which structurally and functionally interact with DNA. Such modifications result in modulation of chromatin conformation around a promoter site leading to transcriptional activation or suppression. In a previous study, results from P1 nuclease analysis strongly suggested that the CMVIE and the CMV sequences compete for the formation of active chromatin [34]. Additionally, other studies provided biochemical evidence that acetylation of histone proteins by histone acetyl transferases (HATs) at specific lysine residues on the amino-terminal tail domains results in active chromatin conformation [35,36]. Moreover, recent evidence linking several transcription factors such as Gcn5, CBP/p300, and TAFII250 to HAT activity strongly suggests a role for acetylation in transcriptional activation. On the other hand, deacetylation of histone proteins at a promoter site by histone deacetylases (HDACs) has been associated with transcriptional suppression. The mechanism is not well understood but several models have been proposed including disruption of the transcription initiation complex, or simply preventing its assembly, or changes in the higher-order structure of chromatin rendering it incompatible with transcription [37].

In fact, HDAC inhibitors have long been used as transcriptional activators. Butyrate, the first identified HDAC inhibitor [38], was used to induce gene expression from type C virus [39] and HIV LTR [40] in infected mammalian cells. However, at the time, it was not known by which mechanism butyrate treatment enhanced LTR-driven gene expression. More recent work demonstrated that HDAC inhibitors can activate transcription from integrated viral promoters [41,42]. Thus, we exploited the use of sodium butyrate for reactivation of retroviral RNA transcription in the SINCMV design. Our results show that treatment of the SINCMV producer cells with 10 mM and 20 mM sodium butyrate for 48 hr resulted in a significant increase in the expression of the three viral RNA transcripts (Figure 1C). Of particular importance is the non-spliced packageable retrovector mRNA that is derived from the upstream 5'CMV promoter that was undetectable in the untreated control cells. Since the level of expression of packageable transcript is rate limiting for viral production, low levels of retroviral transcript expression in untreated producers could have contributed to the reduced viral titer obtained initially. Interestingly, treatment of the SINCMV retroviral producers with increasing doses of sodium butyrate not only reversed transcriptional suppression in the vector, but it also resulted in a significant increase in viral titer (Figure 2B). The effect was dose dependen. Improved titers resulted in a strikingly enhanced gene transfer into A549 lung carcinoma cells (Figure 2C). Furthermore, a histone gel assay confirmed increased histone acetylation in SINCMV producer cells treated with sodium butyrate (Figure 3).

HDAC inhibitors were used in previous studies to boost up production from conventional (non-SIN) retroviral [43-45] and lentiviral [46] vectors. In one study, production of a retroviral vector expressing the normal human cystic fibrosis transmembrane conductance regulator (CFTR) cDNA was significantly enhanced by sodium butyrate treatment of the producer cells with a simultaneous increase in the steady-state levels of LTR-driven full-length retrovector RNA [43]. The authors suggested that the cDNA of CFTR caused an "ill-defined" interference with the LTR transcriptional activity that could have resulted in upfront low titers. However, it is worth noting that their retroviral vector had an internal simian virus40 (SV40) promoter upstream of the neomycin selectable marker. Therefore, it is possible that the low titers associated with this vector could have also resulted from an interference effect between the internal SV40 promoter and the 5'LTR. Moreover, the viral supernatant was harvested from butyrate-containing media that could have lead to increased transgene expression from the integrated viral promoter in transduced cells. Therefore, not all the increase in expression in transduced cells could be attributed to an increase in viral titer and gene transfer.

Since histone hyperacetylation resulting from HDAC inhibition is only one of many cellular changes triggered by sodium butyrate treatment [38], TsA which is a highly specific and more potent HDAC inhibitor [47] was used to determine if histone acetylation is specifically involved in enhanced SINCMV titers. Our results show that treatment of the SINCMV retroviral producer cells with 3 μM TsA resulted in a significant increase in titer indicating that histone deacetylation is indeed implicated in suppression of retroviral RNA transcription in the SINCMV design resulting in reduced titers (Figure 4A). Hereby, we propose a model depicting mechanism of transcriptional suppression in the SINCMV design. It is likely that interferences between strong elements in the internal CMVIE enhancer-promoter and the upstream promoter elements in the 5'LTR lead to the recruitment of histone deacetylases (HDACs) that triggers an inactive chromatin conformation at the promoter sites leading to transcriptional suppression of the retroviral RNA (Figure 4B). However, since the improvement in SINCMV titer with TsA treatment was less marked than that with butyrate, this is highly suggestive that other mechanisms are likely involved.

## Conclusion

In conclusion, our results suggest that SIN retrovectors incorporating strong internal promoters are susceptible to significant transcriptional silencing in packaging cells leading to poor retroviral titers. Treatment of the producer cells with HDAC inhibitors can overcome this blockade suggesting that histone deacetylation is implicated in the mechanism of transcriptional suppression. These findings give us insights for improvement of SIN vector designs with important implications on SIN vector production in many cell and gene therapy applications.

## Methods

### Cell lines and plasmids

pJ6ΩBleo plasmid and 293GPG retroviral packaging cell line [48] were generous gifts from Richard C. Mulligan (Children's Hospital, Boston, MA, USA). 293GPG cells were maintained in 293GPG media [DMEM (Gibco-BRL, Gaithesburg, MD), 10%heat-inactivated FBS (Gibco-BRL) supplemented with 0.3 mg/ml G418 (Mediatech, Herndon, VA), 2 μg/ml puromycin (Sigma, Oakville, ONT), and 1 μg/ml tetracycline (Fisher Scientific, Nepean, ONT)]. A549, a human lung carcinoma cell line, was obtained from the American Type Culture Collection (ATCC, Manassas, VA) and was maintained in DMEM supplemented with 10%heat-inactivated FBS and 1% penicillin-streptomycin.

### SINCMV retrovector design and synthesis

We used a derivative of pLTRGFP [10] to generate the SINCMV design. pLTRGFP contains the cDNA for the enhanced green fluorescent protein (EGFP) reporter and a full-length LTR whose U3 region is derived from MSCV and whose R and U5 regions are derived from pCMMPLZ, a MFG derivative. We derived a self-inactivating vector from pLTRGFP by creating a 311-bp NheI-SacI deletion to the 3'LTR to remove all the enhancers and the CAAT box. The synthesis of SINCMV was as follows. The 655-bp insert encoding for the CMVIE enhancer-promoter was excised by AseI/Klenow and AgeI digest of a shuttle vector that was derived from pEGFP-C1 (CLONTECH, Palo Alto, CA). This insert was ligated into the product of BglII/Klenow and AgeI digest of the NheI-SacI SIN-derivative in order to generate the SINCMV plasmid. Both control SIN and SINCMV vectors incorporate the CMV promoter in the 5'LTR that drives expression in transfected producer cells. Nucleotide sequences of the mutated 3'LTR and the inserted CMVIE promoter were confirmed by DNA sequencing (GenAlyTic Inc., University of Guelph, ONT).

### Generation of the retroviral producers

The retroviral producers were generated by stable transfection of the 293GPG packaging cell line as previously described [10]. In brief, stable producer cells were generated by co-transfection of 5 μg FspI-linearized control SIN vector or SINCMV vector and pJ6ΩBleo plasmid at a 10:1 ratio. Transfected packaging cells were subsequently selected in 293GPG media supplemented with 100 μg/ml Zeocin (Invitrogen, San Diego, CA) for 3-to-4 weeks. Resulting stable polyclonal as well as isolated single clone producer populations were utilized to generate VSV-G pseudotyped retroviral particles. We selected producer clone 4 to perform subsequent experiments with butyrate. TsA experiments were performed on the polyclonal producer population to rule out any clonal effect that may have attributed to increased titers from producer clone 4 with butyrate treatment.

### Treatment of producers with histone deacetylase inhibitors for retroviral production

Working stocks of 1 M sodium butyrate were prepared from concentrated n-Butyric Acid (Acros Organics, NJ) in distilled water, then filtered with 0.2-micron syringe mounted filters (Gelman Sciences, Ann Arbour, MI), and stored at 4°C. Trichostatin A (TsA) stock (BIOMOL Research Laboratories, Inc., PA) was stored at -20°C. The treatment of control SIN or SINCMV producer cells with histone deacetylase inhibitors was as follows. The retroviral producer cells were maintained in 293GPG media in 100-mm tissue culture dishes. At 70 to 80% confluency, the tetracycline-containing media was replaced with complete DMEM to allow for VSV-G expression and subsequently for retroviral production. One day post tetracycline withdrawal, either sodium butyrate in mM or TsA in μM concentrations were added to the cells in complete DMEM media for 48 hr, at 37°C and 5%CO_2_. Afterwards, the drug-containing supernatant was discarded and fresh complete DMEM media was added to the producer cells to harvest retroviral supernatant in 24 hr. All viral supernatants were filtered with 0.45-micron syringe mounted filters (Gelman Sciences) and stored at -20°C.

### Viral titer determination and RCR assay

A549 target cells were plated in 6-well dishes at 4 × 10^4 ^cells per well and allowed to adhere overnight in complete media (DMEM, 10%heat inactivated FBS, and 50 units/ml Pen/Strep) at 37°C and 5%CO_2_. The following day, the overlaying medium was aspirated and replaced with 1 ml per well of serial dilutions in complete DMEM media of the viral sample supplemented with 6 μg/ml Polybrene (Sigma). Target cells were then incubated with the viral dilutions overnight at 37°C and 5%CO_2_. Subsequently, they were washed with 2 ml per well of phosphate-buffered saline and were then expanded in culture in complete DMEM media. Flow cytometry analysis (FACStar sorter, Becton Dickinson, Mountain View, CA) was then performed on these samples within 5-to-10 days following transduction to ascertain retrovector expression and gene transfer efficiency as measured by EGFP fluorescence. The viral titer was calculated from the gene transfer values obtained with each viral dilution and expressed as infectious particles per ml. Viral preparations were devoid of replication competent retrovirus (RCR) as determined by the standard EGFP marker rescue assay performed on null A549 cells with conditioned supernatant collected from transduced A549 cells.

### RNA extraction from SINCMV retroviral producer cells

Total RNA was extracted from stable 293GPG-SINCMV retroviral producer cells using TRIZOL reagent (Gibco-BRL, Gaithersburg, MD) according to the manufacturer's specifications. In brief, cells from 90% confluent 10-cm tissue culture dish were lysed with 1 ml of the TRIZOL solution. RNA was then extracted with 100% chloroform and precipitated with 100% isopropanol at -80°C for over 1 hr. The precipitated RNA was then washed with 75% ethanol, air-dried for 5 min and resuspended in diethylpyrocarbonate (DEPC)-treated water and stored at -80°C.

### Northern blot assay

Samples of 10 μg total RNA in loading buffer were heated at 60°C for 10 min, then loaded onto a 1% agarose-1.1% formaldehyde gel, and electrophoresed in 1X MOPS buffer for 3 hr at 150V. Afterwards, the gel was photographed under UV exposure and the RNA was transferred overnight onto a Hybond™-N nylon membrane optimized for nucleic acid transfer (Amersham Pharmacia Biotech, Buckinghamshire, England) using 20X SSC transfer buffer. The blotted RNA was then UV cross-linked to the membrane and hybridized at 68°C using the ExpressHyb™ Hybridization Solution (Clontech, Palo Alto, CA) with a P^32 ^labeled EGFP probe prepared by the random oligolabelling kit (Amersham Pharmacia Biotech, Piscataway, NJ). The hybridized blot was washed twice at 68°C with 2X SSC/0.1% SDS and 0.2X SSC/0.1% SDS respectively, then exposed to X-ray photographic film (Kodak X-Omat) at -80°C.

### Isolation of histone proteins from SINCMV retroviral producers

The isolation of histone proteins was done with some modifications to a previously described procedure [49]. SINCMV retroviral producer cells were trypsinized from a confluent 100-mm tissue culture dish, washed with PBS, and spun at 1800 rpm for 5 min. The cell pellet was re-suspended and lysed in 1 ml ice-cold Nuclear Buffer (NB) (0.25M sucrose, 0.2M NaCl, 10 mM Tris/HCl – pH 8.0, 2 mM MgCl_2_, 1 mM CaCl_2_, and 1% Triton X-100) supplemented with protease inhibitors (Complete, Mini, EDTA- free, Roche Diagnostics, Mannheim, Germany) and re-spun at 13000 rpm for 1 min to pellet the nuclei. The nuclei-containing pellet was then resuspended and incubated in 100 μl of 0.2M H_2_SO_4 _at 4°C overnight. Afterwards, the insoluble fraction was pelleted at 13000 rpm for 10 min and the supernatant containing the histone proteins was transferred to a clean 1.5 ml tube. The Bio-Rad Protein Assay kit (Bio-Rad Laboratories, Hercules, CA) was used to determine the protein content in the supernatant. Histone proteins were then precipitated using 900 μl ice-cold acetone at -20°C overnight, and air-dried for 5 min after spinning at 13000 rpm for 10 min. Finally, the isolated histone proteins were re-suspended to 5 μg/μl in Acid Urea (AU) sample buffer (8M urea, 10% glycerol, 5% acetic acid, and 2% w/v methyl green dye).

### Acid Urea Triton (AUT) gel electrophoresis

Analysis of histone acetylation was performed using Acid Urea Triton (AUT) gel electrophoresis that was done with little modifications to procedures described previously [49,50]. We used a gel (Mini PROTEAN II-Bio-Rad) that consisted of 12% (w/v) acrylamide, 0.08% bisacrylamide, 5% acetic acid, 8M urea, 6 mM Triton X-100 and polymerized with TEMED and 25% ammonium persulfate (APS). After polymerization, the gel was pre-run for 2 hr in 5% acetic acid buffer at 200V. Then, fresh 5% acetic acid was used and 30 μg of each sample was loaded onto the gel and electrophoresed at 135V- 200V until the bromophenol blue dye migrated out. Finally, the gel was stained for 2 hr with 0.03% Coomassie Brilliant Blue R250 plus 50% ethanol, and 10% acetic acid. In order to visualize the protein bands, the gel was de-stained using 20% ethanol and 10% acetic acid.

### Statistics

Unless otherwise specified, all results are reported as average of three independent experiments ± standard error of the mean. Student T test was applied using Microsoft Excel software.

## Competing interests

The author(s) declare that they have no competing interests.

## Authors' contributions

DJ carried out the cloning and generation of the various vectors, retroviral production and titer assays, transduction and subsequent analysis of target cells, transcriptional assays, data acquisition and analysis, and drafted the manuscript. MC did the work relating to the histone gel assay, helped in the above experiments in tissue culture, and revised the manuscript. PB contributed to the conception of the designs in the study, acquisition of funding, and revised the manuscript. JG had substantial contribution to the conception of the study, the experimental designs, data analysis and interpretation, general supervision of the research, acquisition of funding, and critically revised the manuscript. All authors approved of the final manuscript version.
